# Effect of long-term influx of tertiary treated wastewater on native bacterial communities in a dry valley topsoil: 16S rRNA gene-based metagenomic analysis of composition and functional profile

**DOI:** 10.7717/peerj.15583

**Published:** 2023-06-26

**Authors:** Abdurrahman S. Masrahi

**Affiliations:** Department of Biology, Faculty of Science, Jazan University, Jazan, Saudi Arabia

**Keywords:** Tertiary treated wastewater, Partially treated wastewater, Polluted valley soil, Unpolluted valley soil, Bacterial community composition and function

## Abstract

Although dumping treated wastewater into soil might provide nutrients and organic matter, it can also expose the ecosystem to biological and chemical risks. A vital indication of soil health and quality is the soil microbial community. The current work used next-generation 16S rRNA gene amplicon sequencing to evaluate the effects of the long-term influx of tertiary treated wastewater (TWW) into Wadi Uranah, a dry valley in Makkah city, Saudi Arabia, on native topsoil bacterial community composition and predicted functions. The findings demonstrated that neither the compositions of microbial communities nor their predicted functions using PICRUSt2 differed significantly (*p* > 0.05) between polluted valley soil (PolVS) and unpolluted valley soil (UPVS). Alpha and beta diversity, however, showed that the PolVS samples had a considerably higher level of diversity and variability. Firmicutes, Actinobacteria, Proteobacteria, and Bacteroidetes were the most prevalent phyla in both groups. Noticeable relative variations existed in some metabolic pathways such as cofactor, prosthetic group, electron carrier degradation, aldehyde degradation, and Entner-Doudoroff (ED) pathways. Overall, our findings suggest that because both groups have very similar core microbiomes and functions, the long-term disposal of tertiary TWW into Wadi Uranah may have little to no influence on the composition and function of soil bacterial communities. In addition, the long-term discharge of tertiary TWW after partially treated wastewater’s initial disposal may have helped the native soil microbial community recover.

## Introduction

The disposal of treated wastewater is a global environmental concern since it threatens humans and the environment. Until the mid of 20th century, raw sewage used to be disposed into streams or oceans for dilution and natural purification processes (Spellman & Drinan, 2001). At present, many countries have standards for the discharge of treated wastewater (TWW) originating from wastewater treatment plants (WWTPs), but such standards vary across the world (Libralato, Ghirardini & Avezzù, 2012; Angelakis & Snyder, 2015). Due to the scarcity of freshwater resources and increased population density and demand, the reuse of TWW has become inevitable and common worldwide, especially in arid and semiarid regions of the world (Bixio et al., 2006). Such uses of TWW include but are not limited to, agricultural irrigation (Elbana, Bakr & Elbana, 2017), supplementing drinking water needs (Adewumi, Ilemobade & Van Zyl, 2010), aquaculture (Kumar et al., 2015), and groundwater recharge (Fournier et al., 2016). Releasing and reusing TWW may be beneficial in supplying nutrients and organic matter, but may also introduce biological and chemical hazardous agents such as micropollutants, pathogens, and antibiotic-resistant microbes into the environment (Kümmerer, Dionysiou & Fatta-Kassinos, 2015). Moreover, the consequences of introducing such hazards (*e.g.*, on soils and human health) on the possible disturbance of native soil microbial communities and functions are still poorly understood (Kümmerer, Dionysiou & Fatta-Kassinos, 2015; Saarenheimo et al., 2017). Soil microbial communities are fundamental indicators of soil health and productivity in any ecosystem (Kennedy & Stubbs, 2006; Lopes et al., 2015). Microorganisms have vital roles in terrestrial and aquatic ecosystems, such as mineralization of organic matter, recycling of mineral nutrients and supplying utilizable forms of nutrients for plants, and soil reclamation (Balser, Kinzig & Firestone, 2002; Singh et al., 2019).

The contamination of surface soil and groundwater due to continuous expansion of urban areas is a worrying issue in Saudi Arabia. Makkah is the third largest city in Saudi Arabia, with a population of over 1.5 million (General Authority for Statistics, Kingdom of Saudi Arabia, 2010). The WWTP of Makkah, located southwest of the city, is continuously discharging TWW into the southwest section of Wadi Uranah, a dry valley stretching from southeast to southwest of Makkah toward the Red Sea. The valley’s soil is mainly composed of alluvial deposits, with the surface covered with a layer of eolian sand (Al-Rehaili & Bankhar, 1998). The valley receives small amounts of precipitation annually, mainly during winter. In 1984, the WWTP of Makkah started to dump between 70,000 and 100,000 m^3^ of partially TWW in the southwest section of Wadi Uranah (Bahabri, 2011; Rashed et al., 2020) but may exceed this amount during special seasons in Makkah (the holy month of Ramadan and pilgrimage). In 2012, a new WWTP with a tertiary treatment system was built, with a daily capacity of 375,000 m^3^ (Rashed et al., 2020).

Environmental assessment studies conducted prior to 2012 regarding the contamination of soil and water at Wadi Uranah have indicated the presence of a high concentration of pollutants. For example, a study conducted by Al-Harthi (2001) showed that the TWW could not be used for human consumption or domestic and irrigation purposes due to high concentrations of heavy metals, organic matter, salts, and bacteria. Similarly, Bahabri (2011) also indicated that in both soil and water chemical and microbiological contamination levels exceeded the limits of local and international drinking and irrigation water quality standards. Nevertheless, in a study conducted by El-Solimani (2001), total and fecal coliforms in TWW were very high at the disposal outlet and during the month of pilgrimage but decreased with distance from the outlet during other months. He further suggested that using TWW from this site should be restricted.

Moreover, Al-Rehaili & Bankhar (1998) also have indicated that the microbiological analysis showed that the groundwater is contaminated and, therefore, cannot be used for human consumption.

Recent research has illustrated the safe reuse of tertiary TWW in both soil and plants. In a study conducted by Petousi et al. (2019), irrigation of grapevines with tertiary TWW for 3 years, starting from planting, had very limited effects regarding the risk of microbial and chemical contamination of the vineyard (soil-plant-fruit system), therefore ensuring safe reuse for the community’s health and the environment. In another study, Bakari et al. (2022) reported that the irrigation of strawberries with a 60% diluted tertiary TWW (40%) had resulted in fruits that match the international safety and quality standards. In their study, they reported a lower content of heavy metals than international standard limits (FAO/WHO) and the absence of microbial contamination by bacteria such as fecal coliform. Although the reuse of tertiary TWW can be considered safe, especially in the long-term, it is always recommended to continually monitor soil health to ensure that the reuse of such TWW does not have negative impacts on soil physical, chemical, and biological properties. However, it is important to note that depending on the inherent properties of the soil, the environment, the source of the wastewater, and the efficacy of the reclamation process, irrigation with treated wastewater may have varying effects on the soil (Leogrande et al., 2022).

The microbiological analyses of previous studies conducted in the study site were based on cultural methods and conducted before establishing the new advanced WWTP at Makkah city. Therefore, this raises the need for environmental assessment of the long-term effects of tertiary TWW discharge on native soil microbial communities. Given this background, this study aims to assess the effects of the long-term discharge of tertiary TWW on native soil microbial community composition and functions at Wadi Uranah.

## Materials & Methods

### Soil sample collection

**Figure 1:**
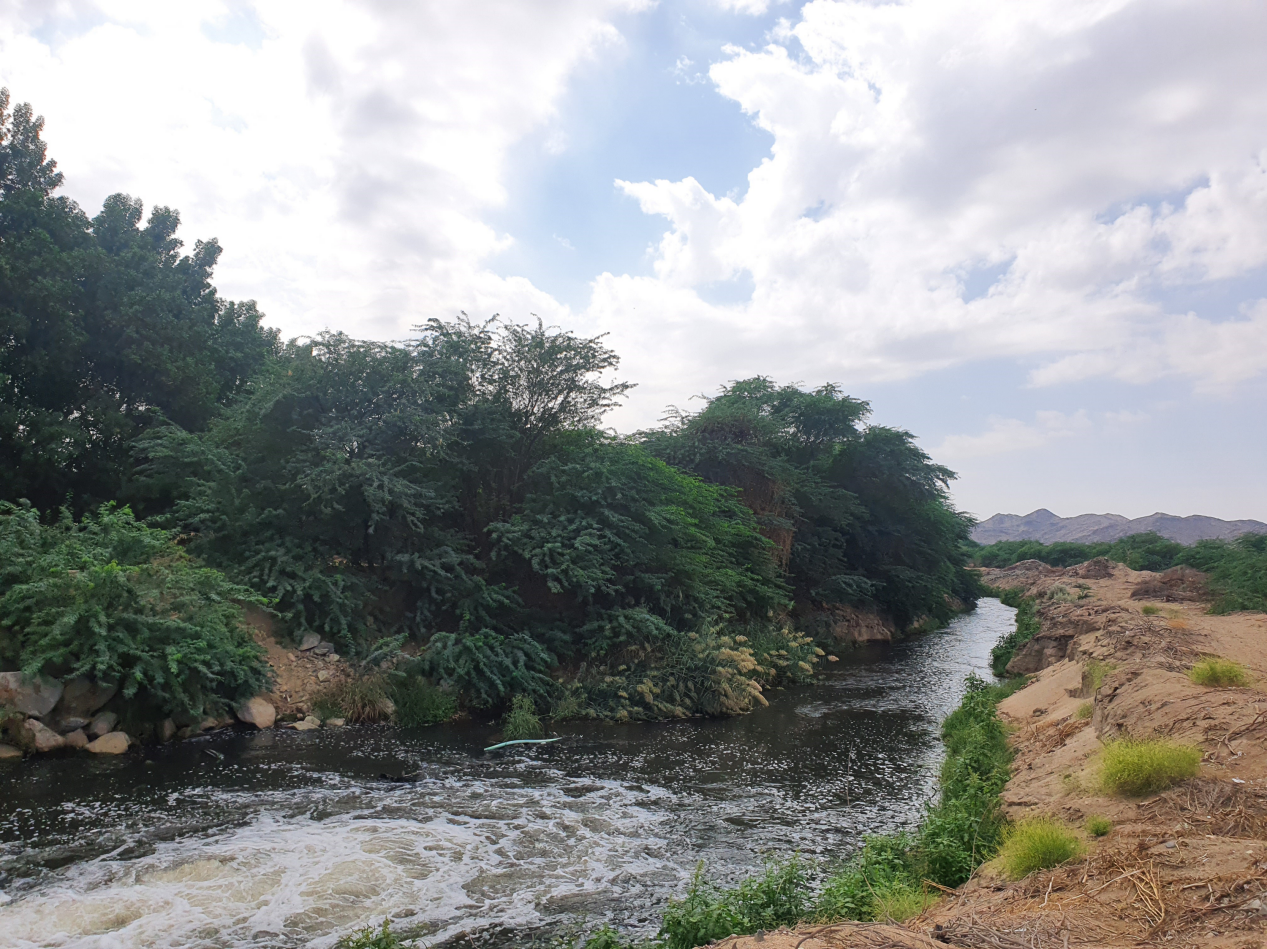
The main outlet of the treated wastewater disposal at Wadi Uranah. Latitude and longitude: 21°15′03.7″N and 39°42′34.1″E. Photograph by the author.

**Figure 2:**
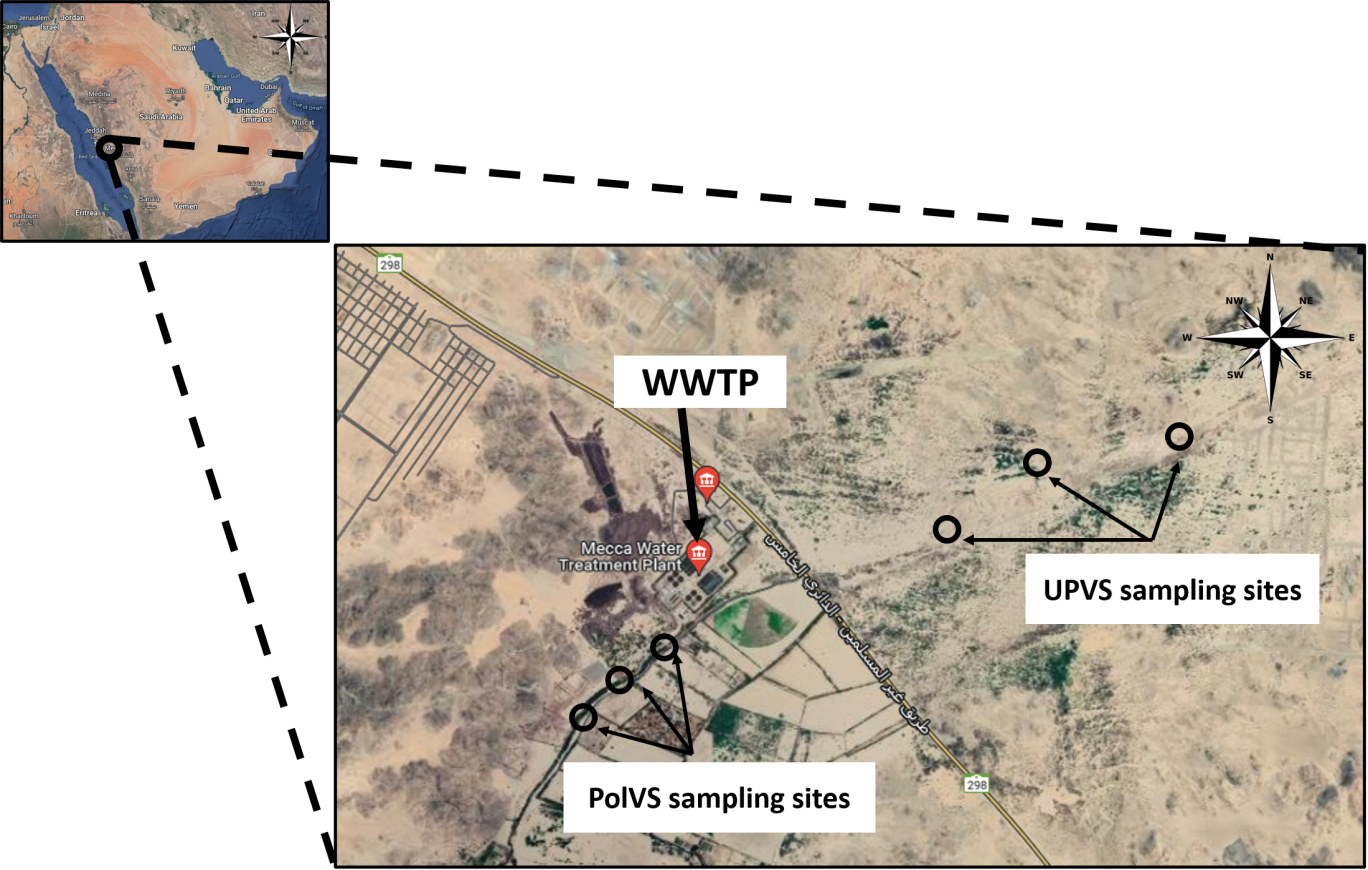
Location map of the sampling sites near the wastewater treatment plant (WWTP) at Wadi Uranah. UPVS: unpolluted valley soil. PolVS: polluted valley soil.

Two areas along Wadi Uranah near Makkah city’s WWTP (21°15′19.8″N latitude and 39°42′41.3″E longitude) were chosen for soil sampling. Each area was divided into three sub-locations to represent three replicates. The first area is east of the WWTP (unpolluted valley soils) and was chosen as a reference for the native microbial community. The second area is located west of the WWTP alongside the main outlet disposal stream of the treated wastewater (TWW) (polluted valley soils) (Fig. 1). A total of six soil samples (0–15 cm) were collected from the two areas, with three replicates for each area. Soil samples from the polluted area were collected close to the water edge (soil/water interface) from both sides. Each replicate consisted of 3 subsamples pooled together, with a distance between the subsamples of approximately 2–3 m. The six soil sampling locations are illustrated in Fig. 2. The soil sampling was carried out in November 2021.

### Soil physical and chemical properties

The soil texture from both locations was determined using a hydrometer (Bouyoucos, 1962). The percentage of soil organic matter (SOM) was determined by the wet oxidation method (Walkley & Black, 1934). Soil pH, electrical conductivity (EC), and total dissolved solids (TDS) were determined with a pH meter using a 1:2.5 soil: water suspension. Soil moisture content was determined by oven-drying method at 105 °C. Water soluble NO_3_^−^, NH_4_^+^, and SO_4_^2−^ were analyzed spectrophotometrically. Total phosphorus in the soil was determined using a digestion and spectrophotometric analysis method (APHA 4500-P E) (Rice et al., 2012).

### Soil microbial DNA extraction

Soil microbial community DNA was extracted using a DNeasy PowerSoil Pro Kit (Qiagen, Germany) following the manufacturer’s instructions. Following extraction, DNA samples were quantified to detect DNA quality using a NanoDrop 8000 spectrophotometer (Thermo-Fisher Scientific Inc., Wilmington, DE, USA) and then stored at −20 °C for later analysis.

### 16S rRNA gene amplicon sequencing

The DNA samples were shipped to Beijing Genome Institute (BGI) (Shenzhen, China) for amplification, library construction and next-generation sequencing. The PCR system was configured using 30 ng of qualified DNA template along with fusion primers for PCR amplification. The V3-V4 region of the bacterial 16S rRNA gene was amplified using the following PCR primers: 319 F (5′-ACTCCTACGGGAGGCAGCAG-3′) and 806 R (5′-GGACTACHVGGGTWTCTAAT-3′). The PCR program was as follows: initial denaturation at 98 °C for 3 min, 30 cycles of denaturation at 98 °C for 45 s, annealing at 55 °C for 45 s, and extension at 72 °C for 45 s, and a final extension at 72 °C for 7 min. All PCR products were purified by Agencourt AMPure XP beads, dissolved in an elution buffer, and eventually labeled to finish library construction. Library size and concentration were detected by Agilent 2100 Bioanalyzer (Agilent, Santa Clara, CA, USA) before being sequenced on an Illumina HiSeq 2500 platform (Illumina, San Diego, CA, USA) according to standard Illumina pipelines to generate 2 × 300 bp paired-end reads. The raw data were filtered to generate high-quality clean reads as follows: truncate reads with average Phred quality values lower than 20 over a 25 bp sliding window were truncated. Reads were removed if their length was 75% lower than the original lengths after truncation. Low-complexity, N-containing, and contaminated reads by adapter sequences were also removed (Martin, 2011; Fadrosh et al., 2014). All raw sequences were deposited at the National Center for Biotechnology Information (NCBI) under the SRA accession ID PRJNA915583.

### Bioinformatics, functional prediction, and statistical data analysis

Following removing of low-quality and ambiguous bases to filter raw reads, paired-end reads were linked to tags using the Fast Length Adjustment of Short reads program (FLASH, v1.2.11) (Magoč & Salzberg, 2011). The tags were then clustered into operational taxonomic units (OTUs) with a cutoff of 97% threshold using UPARSE software (v7.0.1090) (Caporaso et al., 2010), and chimera sequences were compared with the Gold database using UCHIME (v4.2.40) (Edgar, 2010). The OTUs sequences were taxonomically assigned using Ribosomal Database Project (RDP) classifier (v2.2) (Cole et al., 2014), with a confidence threshold of 0.6, and trained on the Greengenes database (v2.01305) in QIIME (v1.8) (Schloss et al., 2009). All tags were mapped to OTU representative sequences using USEARCH GLOBAL to obtain OTU abundance statistics for each sample.

Nonparametric statistics (Wilcoxon rank-sum test) were used to compare abundance of OTU, bacterial communities, alpha and beta diversity between the groups in USEARCH. Alpha diversity was assessed by observed species, Chao, ACE, and Shannon’s diversity indices using MOTHUR (v1.31.2), with boxplots generated using R (v3.2.1). Beta diversity analyses was performed using weighted UniFrac distance as implemented in QIIME (v1.8.0), and plotted *via* principal coordinate analysis (PCoA) using R (v3.2.1). Venn plots of OTUs was plotted using the “Venn diagram” package in R (v3.1.1; R Core Team, 2015).

The potential metabolic function of bacterial communities for UPVS and PolVS groups was examined using Phylogenetic Investigation of Communities by Reconstruction of Unobserved States 2 (PICRUSt2) (Douglas et al., 2020). Representative sequences were inserted into an existing phylogenetic tree, and the best position of placed OTUs was determined in the reference phylogenetic tree using EPA-NG (Barbera et al., 2019). Using Castor–a hidden state prediction (HSP) tool (Louca & Doebeli, 2018), gene families were predicted and subsequently, inferred into MetaCyc pathways using MinPath (Ye & Doak, 2009).

Differences in the average relative abundances of taxa at genus and species levels and the different functions among groups were analyzed using Wilcox test. *p* values of <0.05 were considered statistically significant.

## Results

### Statistics of 16S rRNA amplicon sequence datasets

**Figure 3:**
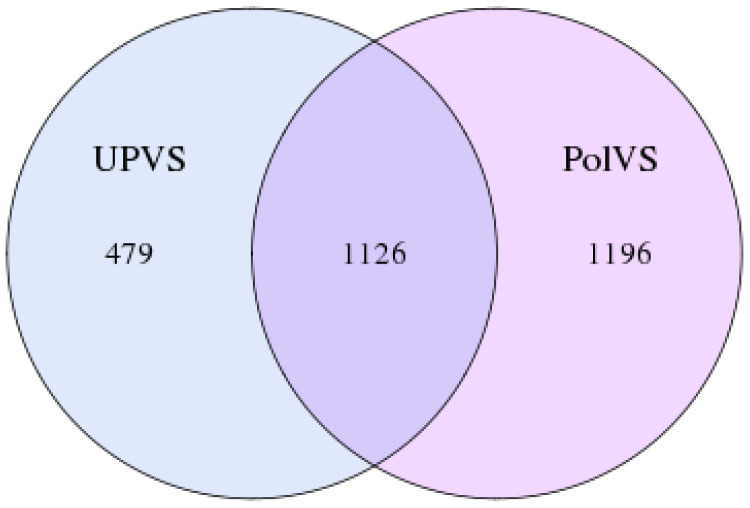
Venn diagram showing unique and shared OTUs of the two groups of samples. PolVS: polluted valley soil. UPVS: unpolluted valley soil.

In this study, the 16S rRNA bacterial community composition of six soil samples (physical and chemical properties are listed in Table 1) belonging to two groups was analyzed using Illumina HiSeq. Statistics of the raw data are shown in Table 2. The average sequence length/read was 298 bp across the different samples ranging from 293 to 300 pb, generating 408,235 clean sequence reads across all samples. A total of 208,220 tag numbers were generated across all samples, with average read number of 36,180 per polluted valley soil (PolVS) samples comparing with 33,226 tags per unpolluted valley soil (UPVS) samples. All sequences were divided into 7,284 OTUs based on a 97% similarity level, with average of 1,293 OTUs per PolVS comparing with 1,134 OTUs per UPVS (Table 2). The Venn diagram showing unique and shared OTUs of the two group of samples is reported in Fig. 3.

**Table 1:** Physical and chemical properties of polluted valley soil (PolVS) and unpolluted valley soil (UPVS).

**Property**	**UPVS**	**PolVS**
Sand (%)	94	98
Silt (%)	2	0
Clay (%)	4	2
Soil moisture content (%)	0.4	13
SOM (%)	>0.5	>0.5
pH	7.13	6.65
EC (mS/cm)	1.16	3.98
TDS	148	509
Total phosphorus (ppm)	552	874
NO_3_^−^ (ppm)	0.02	0.03
SO_4_^2−^ (ppm)	42	256
NH_4_^+^ (ppm)	2	2

**Notes.**

SOM soil organic matter EC electrical conductivity TDS total dissolved solids

**Table 2:** Data generated from the 16S rRNA deep sequencing for soil microbiomes collected from polluted valley soil (PolVS) and unpolluted valley soil (UPVS) near Makkah city wastewater treatment plant (WWTP).

Sample name	Read length (bp)	Raw reads	Clean reads	% Read utilization	Tag number	OTU number
Pol_VS1	300:300	70104	67704	96.58	39558	1976
Pol_VS2	299:300	70148	67676	96.48	35772	927
Pol_VS3	299:299	70326	67871	96.51	33212	977
UP_VS1	296:300	70513	68106	96.59	32318	898
UP_VS2	294:300	70941	68385	96.4	35048	1304
UP_VS3	293:300	71059	68493	96.39	32312	1202

### Soil bacterial community diversity and composition

**Figure 4:**
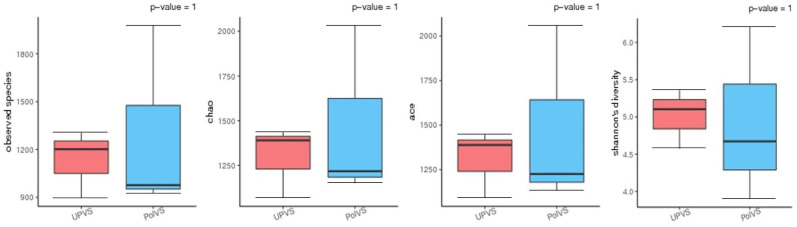
Alpha diversity analysis between polluted valley soil (PolVS) and unpolluted valley soil (UPVS) groups. The five lines from the bottom to the top are the minimum, first quartile, median, third quartile, and maximum, respectively. *P*-values are shown above each graph (*α* = 0.05).

**Figure 5:**
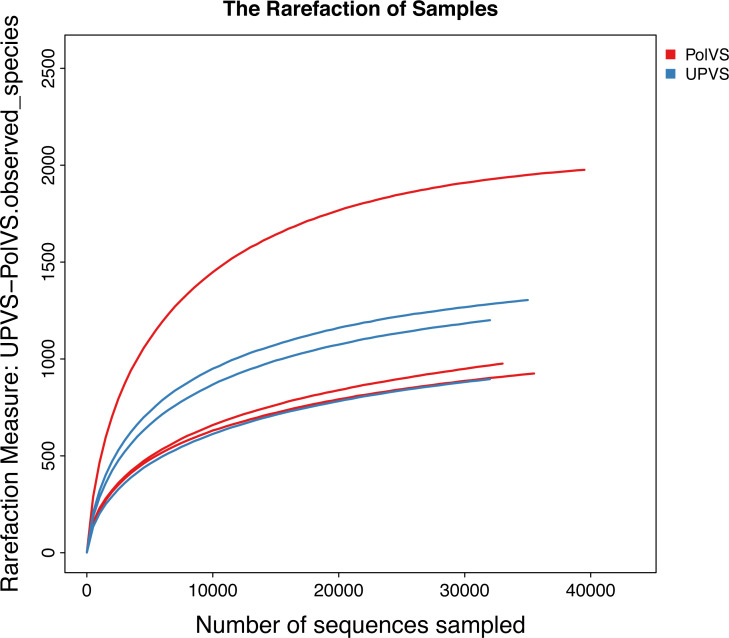
Rarefaction curves based on observed species value of polluted (PolVS) and unpolluted (UPVS) valley soils.

**Figure 6:**
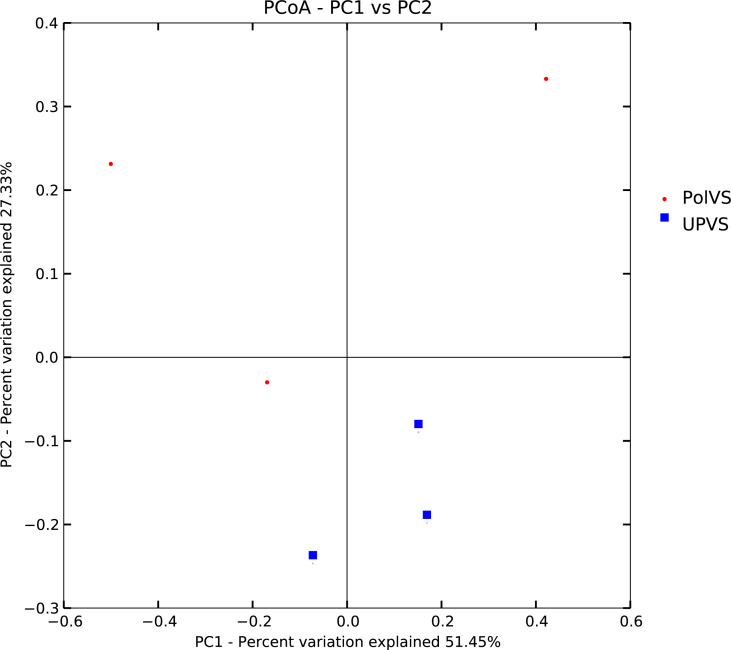
Plot of principal coordinate analysis (PCoA) showing beta diversity (weighted UniFrac distance) of soil bacterial microbiomes of the two groups of samples. PolVS: polluted valley soil. UPVS: unpolluted valley soil.

Based on alpha diversity metrics (Fig. 4), no significant differences were found between PolVS and UPVS groups. Nevertheless, it can be noticed that the PolVS group had relatively higher richness based on observed species, Shannon, Chao, and ACE indices. The rarefaction curves based on observed spices (Fig. 5) tends to be relatively smooth, however, further sequencing might have generated more OTUs in each sample. Principal coordinate analysis (PCoA) was done to describe differences within and among the two groups (Fig. 6). Plot of PCoA did not show a complete separation of microbiome diversity among groups. Diversity within samples of PolVS showed high variability with distribution in both negative and positive directions of PC1 and PC2. On the other hand, while diversity within UPVS samples was distributed in both the negative and positive direction of PC1, they were only located in the negative direction of PC2.

**Figure 7:**
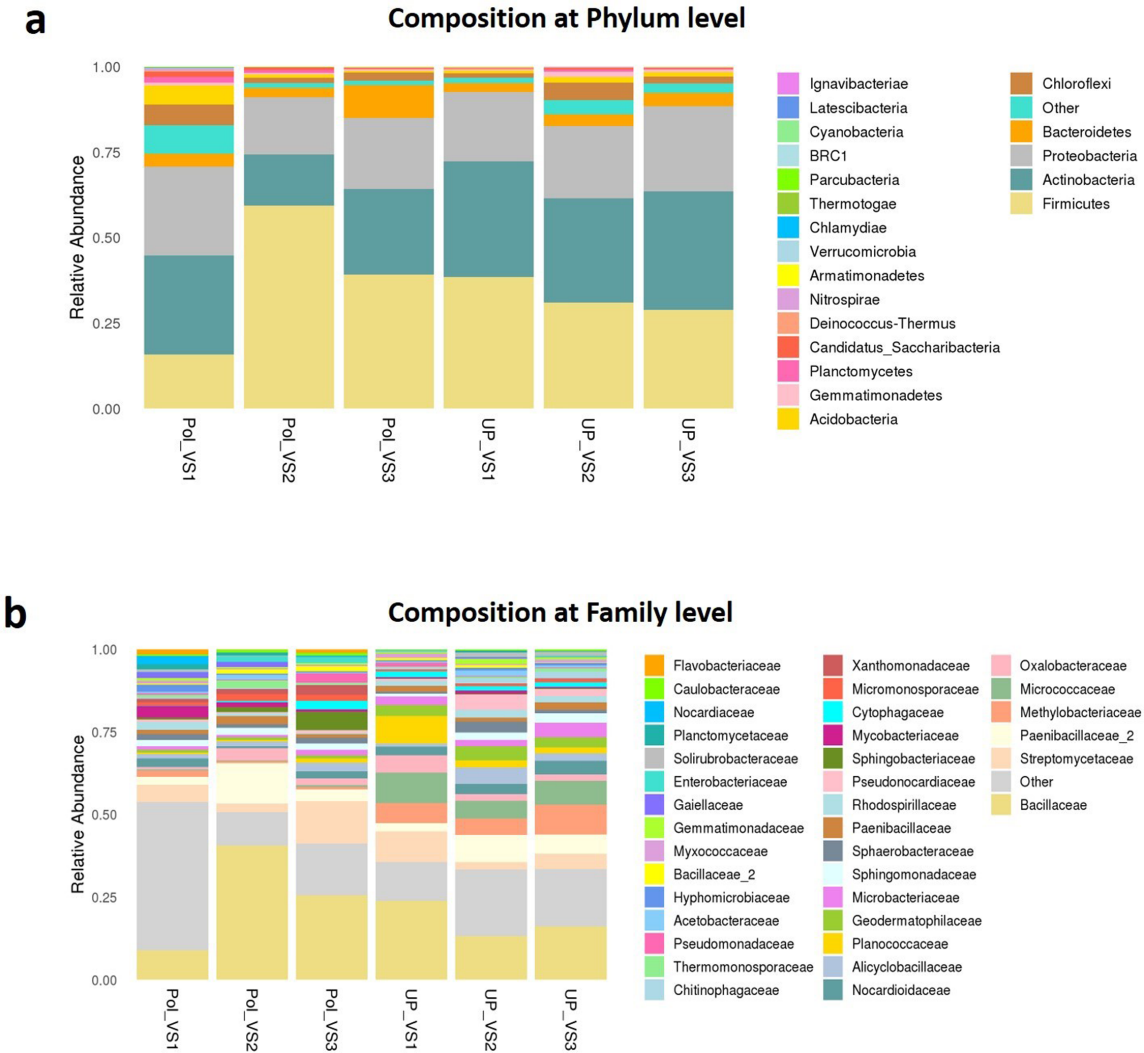
Bacterial community composition of polluted (PolVS) and unpolluted (UPVS) valley soils. (A) Phylum level distribution of dominant soil bacterial taxa in PolVS and UPVS samples. (B) Family level distribution of dominant soil bacterial taxa in PolVS and UPVS samples.

Bacterial community composition at phylum and family levels is reported in Fig. 7. At phylum level, a total of 25 phyla were identified. Actinobacteria was the most abundant phyla by average relative abundance (32.98%) across samples of UPVS, followed by Firmicutes (32.71%), Proteobacteria (22%), and Bacteroidetes (3.3%). On the other hand, the average relative abundance of most abundant phyla across PolVS samples were as follows: Firmicutes (37.3%), Actinobacteria (23.1%), Proteobacteria (21.3%), and Bacteroidetes (5.2%). At the family level, a total of 36 families were identified. Bacillaceae (17.62%), Micrococcaceae (7.2%), Methylobacteriaceae (6.64%), and Paenibacillaceae_2 (5.58%) were the most abundant families across UPVS samples based on average relative abundance, while Bacillaceae (24.44%), Streptomycetaceae (6.69%), Paenibacillaceae_2 (5.95), and Methylobacteriaceae (1.12%) were the most abundant families for PolVS samples. In the case of Micrococcaceae, its relative abundance dropped to 0.41% in the PolVS group of samples. Streptomycetaceae had a relatively similar abundance (5.3%) to that in the UPVS group.

**Figure 8:**
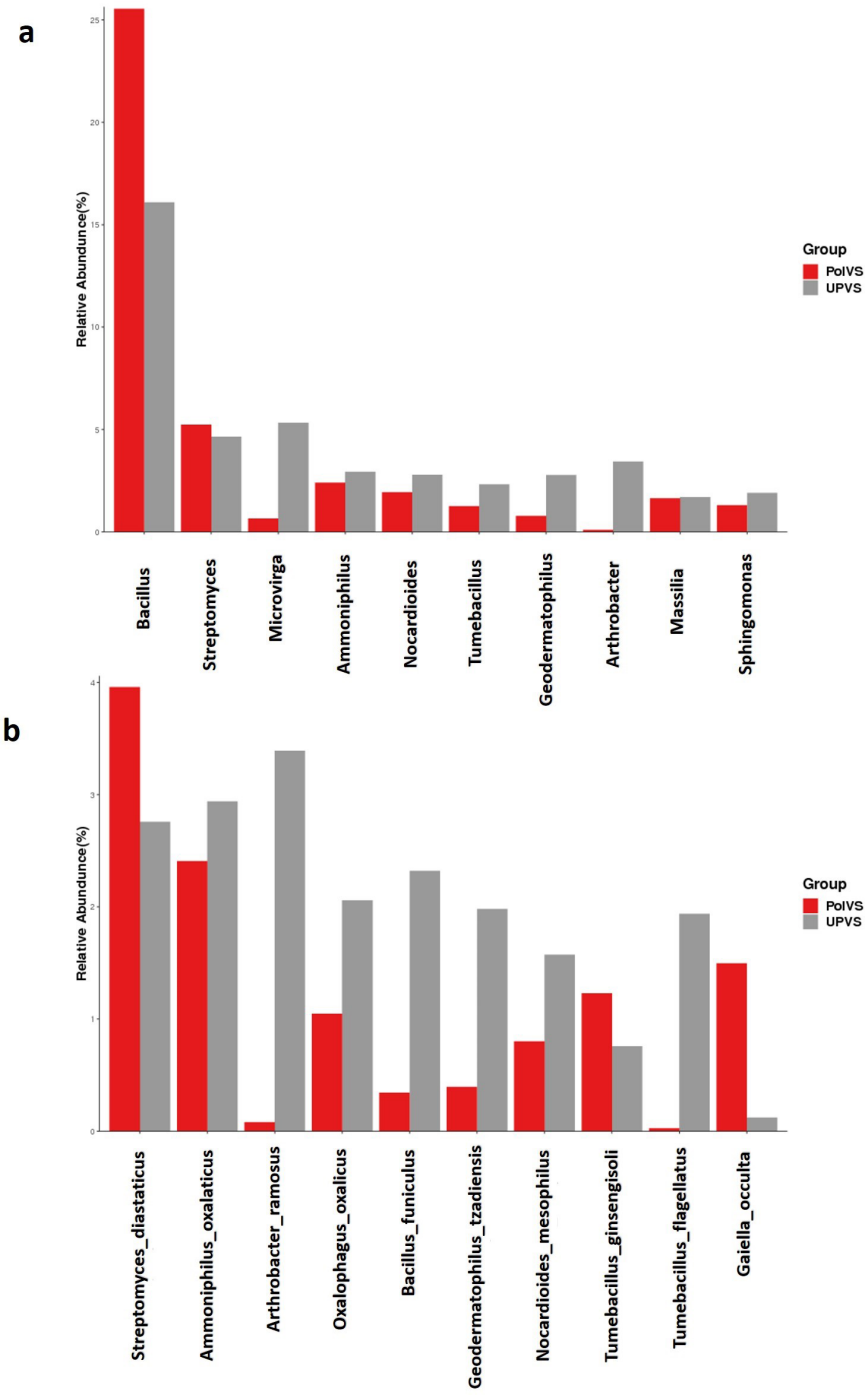
Relative abundance of top 10 differential genera (A) and species (B) among groups. PolVS: polluted valley soil. UPVS: unpolluted valley soil. Significance of the difference test, if any, are marked with an asterisk (*) at the top of the histogram.

Although no significant differences were found (*p* values<0.05) the relative abundances of the top 10 differential genera and species among both groups are shown in Fig. 8. The UPVS samples had slightly higher relative abundance of eight genera, with top three being *Microvirga*, *Arthrobacter,* and *Geodermatophilus*, while *Bacillus* was noticeably higher in PolVS samples. At the species level, we noticed seven genera were higher in UPVS samples, with the top three being *Arthrobacter ramosus*, *Tumebacillus flagellates*, and *Bacillus funiculus*. However, *Streptomyces diastaticus*, *Gaiella occulta*, and *Tumebacillus ginsengisoli* were slightly higher in PolVS samples.

### Functional prediction analysis of bacterial community

**Figure 9:**
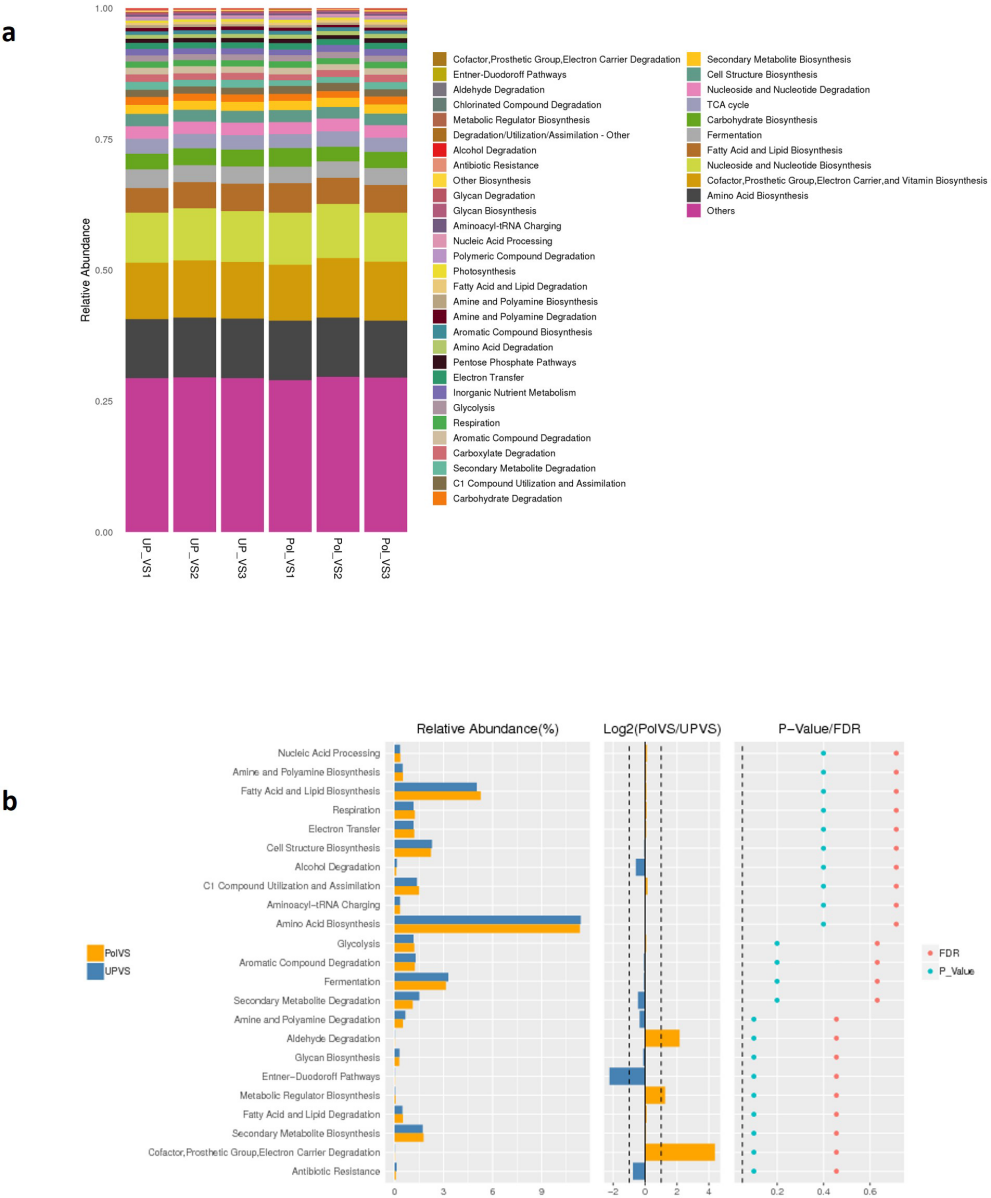
Predicted functions. (A) Barplot of predicted functions showing the relative abundance of MetaCyc pathway level 2 in UPVS (unpolluted valley soil) and PolVS (polluted valley soil) groups. “Others” indicates a pathway that does not contain level 2 information. (B) Results of Wilcox test of top 23 differential functions among groups; barplot of differential relative abundance pathways are shown in the left; log2 values of the ratio of average relative abundance of both groups are shown in the middle; *p*-values and FDR-adjusted *p*-values calculated from Wilcoxon test are shown on the right (*α* = 0.05).

Predicted functional profiling using PICRUSt2 resulted in a total of 2208 KEGG orthologs (enzymes), which were collapsed into 41 MetaCyc pathways level 2 (Fig. 9A). The results showed no significant differences between UPVS and PolVS samples (*p* > 0.05) in all 41 pathways. These 41 pathways were categorized into 8 pathway classes: (1) biosynthesis, (2) superpathways, (3) degradation/utilization/assimilation, 4) generation of precursor metabolite and energy, (5) metabolic clusters, (6) glycan pathways, (7) detoxification, (8) macromolecule modification. Based on Wilcox test, the top 23 differential functions among groups (Fig. 9 b) showed noticeable variation in the abundance of some metabolic pathways such as aldehyde degradation, Entner-Doudoroff (ED), and cofactor, prosthetic group, electron carrier degradation pathways.

## Discussion

Soil is considered the most complex biomaterial on earth, particularly the complexity of soil-microbe interactions that provide vital processes in natural environments, agriculture, and waste management (Young & Crawford, 2004). However, soil microbial community structure and functions can be influenced by multiple biotic and abiotic factors, such as soil pH, soil texture, SOM, nutrient inputs, type of pollutants, land use history, and vegetation cover, among others (Lopes et al., 2015). In this study, deep sequencing of V3-V4 region of the bacterial 16S rRNA gene revealed no significant differences in the overall soil bacterial community diversity and composition between UPVS and PolVS groups. However, higher diversity within PolVS samples was noticeable compared to UPVS samples. Overall, the most abundant phyla in both groups, were Actinobacteria, Firmicutes, Proteobacteria, and Bacteroidetes. These four phyla have been described as dominant in soils (Janssen, 2006). Although the last three phyla showed relatively equal abundances in our study in both groups, we noticed relatively higher abundance of Actinobacteria in the UPVS group (32.98%) compared to the PolVS group (23.1%).

Similar results were also reported in recent studies. For example, in a study conducted by Frenk, Hadar & Minz (2014), the relative abundance of Actinobacteria in semiarid Mediterranean soil was decreased when irrigated with TWW, along with an increase in the relative abundance of Gammaproteobacteria compared to soil irrigated with fresh water (FW). However, they reported that the reduction of Actinobacteria relative abundance was only during the irrigation seasons. However, at the end of three consecutive years, highly similar microbial community composition was observed in all samples of both treatments. As a result, they suggested that Actinobacteria could serve as a marker for healthy and natural microflora in arid and semiarid soils.

Moreover, Ibekwe, Gonzalez-Rubio & Suarez (2018) also reported a decrease in relative abundance of Actinobacteria irrigated with TWW compared to freshwater irrigation. However, they found no significant difference in microbial community composition between TWW and FW irrigations. Actinobacteria have been reported as dominant phyla in arid and semiarid regions (Lauber et al., 2009; Saul-Tcherkas & Steinberger, 2011) and among the microbial community in desert soils (Bao-Zhan et al., 2012; Fierer et al., 2012). The reduction in Actinobacteria abundance in our study could be related to the fact that most members of this phylum are slow-growing microorganisms (K-strategists) (Lebeau, 2011; Brzeszcz et al., 2016). On the other hand, phyla such as Firmicutes are highly resistant to environmental stresses and can quickly outgrow other microorganisms (Sass & Parkes, 2011). However, some studies have linked the reduction in Actinobacteria abundance in the soil to increased SOM (Peacock et al., 2001; Fierer, Bradford & Jackson, 2007; Philippot et al., 2009). In our study, both locations (UPVS and PolVS) had very low SOM, which could be related to the very high sand %. In a study conducted by Jueschke et al. (2008), the authors reported low SOC in different soils with long-term irrigation with TWW compared to FW irrigation. They suggested that the exhausted carbon pool in the soil could be related to the continued priming action by the TWW, causing an intensification of microbial activity. It worth mentioning that, in most of the soils analyzed in their study, % of sand was high (60–89%). The above reasoning may explain the low SOM for the polluted soil samples in our study, which the long-term flow of TWW stream could have influenced.

Although these studies agree with our study, other studies that evaluated the impact of long-term irrigation with TWW on the soil microbial community have shown contrary results. For example, Dang et al. (2019) reported that long-term irrigation with TWW significantly influenced soil microbial community composition at different depths, with increased sensitivity in subsoil compared to surface soil. Also, Zolti et al. (2019) reported a significant change in soil and plant-associated microbial communities regarding activity level, composition and alpha diversity. However, in term of long-term effects, recent research reported that soil texture and properties were the main driving factors in shaping soil microbial community, but not irrigation with TWW (Zolti et al., 2019; Obayomi et al., 2021).

Regarding family level in our study, the relative abundance of Bacillaceae increased from 17.6% in the UPVS group to 24.4% in the PolVS group. Many bacterial species in the family Bacillaceae possess genes involved in adaptive functions such as spore-forming, antibiotic-resistance, and genes for both aerobic and anaerobic growth capabilities (Mandic-Mulec, Stefanic & van Elsas, 2016; Tan et al., 2021). Hence, this may explain the higher abundance of Bacillaceae in the PolVS group. In the case of Micrococcaceae, its relative abundance showed a substantial decrease in PolVS (0.41%) compared to UPVS (7.2%). Thus, this could be related to the lower abundance of the phylum Actinobacteria found in PolVS since Micrococcaceae is a member of the order Micrococcales that possess the highest diversity within Actinobacteria (Alvarez et al., 2017). Furthermore, the relative abundance of the family Methylobacteriaceae dropped from 6.64% in the UPVS group to 1.12% in the PolVS group. The reduced abundance of Methylobacteriaceae in wastewater has been reported in a recent study (Carles et al., 2022) focusing on the impact of wastewater microorganisms on the diversity and functions of periphyton. Their study suggested that the lower abundance of Methylobacteriaceae is due to their known associations with microalgae, which wastewater microorganisms might negatively influence in terms of abundance (Carles et al., 2022).

The current study found no significant differences between UPVS and PolVS among the top 10 differential genera and species. However, some of these genera and species showed relatively high variations between the two groups. The genus *Bacillus* was relatively higher in PolVS compared to UPVS, while *Microvirga* and *Arthrobacter* (specifically here *Arthrobacter ramosus*) where noticeably higher in UPVS compared to PolVS. *Bacillus* is the largest genus within the family of Bacillaceae (Mandic-Mulec, Stefanic & van Elsas, 2016), which could explain the relatively higher abundance of Bacillaceae in PolVS compared to UPVS. On the other hand, *Arthrobacter* and *Microvirga* are aerobic bacteria known to be sensitive to low pH and predominate in alkaline soils (pH >7.5) (Gobbetti & Rizzello, 2014; Msaddak et al., 2017). Consequently, this could explain their relatively lower abundances in PolVS soil due to its acidic pH (6.65) compared to UPVS soils having a pH of 7.13. Besides *Arthrobacter ramosus*, *Tumebacillus flagellates* was slightly higher in UPVS compared to PolVS. This species was first isolated in 2013 from cassava wastewater (Wang et al., 2013). However, since this species was found in both sampling locations (polluted and unpolluted) in our study, it appeared to be a part of the native bacterial community in the area.

Bearing in mind the high variations of soil chemical properties between both locations in our study, predicted functions based on MetaCyc pathway level 2 of both microbial communities revealed no significant differences. Although microbial communities in both groups showed highly similar potential metabolic functions, some metabolic pathways showed relative variations between both groups, such as the aldehyde degradation pathway, the Entner-Doudoroff (ED) pathway, and the cofactor, prosthetic group, electron carrier degradation pathway. Aldehyde groups are well known to possess antimicrobial activity (Zi et al., 2018), and since wastewater is considered a reservoir for the dissemination of organic micropollutants (Kanaujiya et al., 2019) and antibiotic resistant microbes into the environment (Soni et al., 2022), it is expected that the PolVS group would possess higher abundance of organic micropollutant degrading bacteria compared to the UPVS group. The Entner-Doudoroff (ED) pathway is another glycolytic process that is common in aerobic bacteria since it operates under aerobic condition as well as phosphate starvation (Gupta et al., 2021). Thus, this may explain the relative higher abundance of this function in the UPVS group compared to the PolVS since the later had a much higher soil moisture content and relatively higher soil total phosphorus. Higher soil moisture content and presence of pollutants are favorable conditions for microbial anaerobic degradation processes, which could explain the relatively higher abundance of cofactor, prosthetic group, electron carrier degradation pathway in the PolVS group (Li et al., 2022).

The vitality of microbial functional roles in the environment are well known. The most important aspect of these communities concerning soil quality and fertility is their biogeochemical activity. However, the extent and rate of effects of TWW on biogeochemical transformation have yet to be deeply explored (Lopes et al., 2015). However, some studies on specific microbial processes affected by irrigation with TWW have been reported. For example, the nitrification process has increased during the irrigation season with TWW (Kijne, 2011). Moreover, in a study conducted by Oved et al. (2001), significant shift in the composition of ammonia-oxidizing bacteria (AOB) was found in soil irrigated with wastewater effluent compared to irrigation with fertilizer-amended water. Nevertheless, they found no significant changes in community function between wastewater effluent and fertilizer-amended water-irrigated soils. In addition, Frenk, Hadar & Minz (2014) reported that the disturbance of microbial community by TWW is only in the short term. However, they return to the baseline state of core microbial community composition in the long term. Similar results were also reported by Elifantz et al. (2011), where microbial community activity showed a re-bounce to a baseline state after each irrigation season and during winter time in a long-term study using TWW. These studies and our study may support the concept of functional redundancy, meaning that regardless of changes in microbial community composition, the same reactions will be undertaken (Oved et al., 2001; Gelsomino et al., 2006; Lopes et al., 2015).

## Conclusions

In conclusion, both groups share relatively similar core microbiomes and functions. Therefore, our results suggest that the impact of the long-term discharge of tertiary TWW into Wadi Uranah may have minimal or no effects on soil bacterial community composition and function. Based on previous studies that reported a significantly higher level of contaminants during the disposal of partially treated wastewater, the long-term disposal of high-quality tertiary TWW into Wadi Uranah may have contributed to the recovery of the native soil microbial community. Even though both soils in our study highly varied in terms of chemical properties, the high % of sand and low % of SOM could be significant factors in minimizing changes in soil bacterial community composition and functions in the study area. Recent studies have also reported similar observations (Zolti et al., 2019; Obayomi et al., 2021). Since the discharge of tertiary TWW into Wadi Uranah may have no harmful effects on soil microbiological quality in the long term, the increase in population during special seasons in the city of Makkah (the holy month of Ramadan and pilgrimage) may cause temporal changes to soil microbial community composition and functions due to the large quantity of discharged TWW in such seasons.

## Additional Information and Declarations

### Data Availability

The following information was supplied regarding data availability: The sequences are available at the National Center for Biotechnology Information (NCBI SRA): PRJNA915583.
